# MicroRNA-9 as Potential Biomarker for Breast Cancer Local Recurrence and Tumor Estrogen Receptor Status

**DOI:** 10.1371/journal.pone.0039011

**Published:** 2012-06-18

**Authors:** Xin Zhou, Catalin Marian, Kepher H. Makambi, Ourania Kosti, Bhaskar V. S. Kallakury, Christopher A. Loffredo, Yun-Ling Zheng

**Affiliations:** 1 Cancer Prevention and Control Program, Lombardi Comprehensive Cancer Center, Georgetown University, Washington, District of Columbia, United States of America; 2 Department of Pathology, Lombardi Comprehensive Cancer Center, Georgetown University, Washington, District of Columbia, United States of America; The University of Texas MD Anderson Cancer Center, United States of America

## Abstract

MicroRNAs (miRs) are small, non-protein coding transcripts involved in many cellular functions. Many miRs have emerged as important cancer biomarkers. In the present study, we investigated whether miR levels in breast tumors are predictive of breast cancer local recurrence (LR). Sixty-eight women who were diagnosed with breast cancer at the Lombardi Comprehensive Cancer Center were included in this study. Breast cancer patients with LR and those without LR were matched on year of surgery, age at diagnosis, and type of surgery. Candidate miRs were identified by screening the expression levels of 754 human miRs using miR arrays in 16 breast tumor samples from 8 cases with LR and 8 cases without LR. Eight candidate miRs that showed significant differences between tumors with and without LR were further verified in 52 tumor samples using real-time PCR. Higher expression of miR-9 was significantly associated with breast cancer LR in all cases as well as the subset of estrogen receptor (ER) positive cases (p = 0.02). The AUCs (Area Under Curve) of receiver operating characteristic (ROC) curves of miR-9 for all tumors and ER positive tumors are 0.68 (p = 0.02) and 0.69 (p = 0.02), respectively. In ER positive cases, Kaplan-Meier analysis showed that patients with lower miR-9 levels had significantly better 10-year LR-free survival (67.9% vs 30.8%, p = 0.02). Expression levels of miR-9 and another miR candidate, miR-375, were also strongly associated with ER status (p<0.001 for both). The potential of miR-9 as a biomarker for LR warrants further investigation with larger sample size.

## Introduction

Widespread screening strategies through mammography have led to the detection of breast cancer at early stages with a clear positive impact on treatment outcome and patient survival [1]. Several treatment options are available for early stage breast cancer patients, including breast conserving surgery (BCS) followed by radiation therapy and/or adjuvant systemic therapy where appropriate [2], [3]. However, patients who elect breast conserving surgical treatments have a higher risk of developing local recurrence (LR) than patients treated by radical mastectomy [4]. Moreover, a subset of patients will develop LR despite receiving radiation therapy [5]. Therefore, the discovery and characterization of biomarkers for predicting LR could have an impact on the choice of optimal treatment regimes and clinical management of breast cancer.

Several factors associated with higher recurrence risk have been described. Well established risk factors include tumor involved surgical margins, tumor multicentricity, and younger age at diagnosis [6]–[8]. Additional risk factors, albeit with limited predictive power, have been identified such as a family history of breast cancer and other tumor characteristics (e.g. extensive intraductal component, lobular carcinoma, estrogen receptor negative, lymph node invasion) [9], [10]. However, a clear-cut set of biomarkers that can accurately predict LR is still to be identified.

Molecular classification of breast cancer based on gene expression profiling, including with commercially available assays (OncotypeDX, MammaPrint), has been shown to distinguish between distinct breast cancer subtypes that have different prognosis, but their reliability in predicting LR has not been confirmed [11]–[16]. Recent studies suggest that molecular classification based on miR expression profiles is capable of accurately distinguishing breast cancer subtypes [17], [18].

miRs are small non-coding RNA molecules that regulate the activity of specific mRNA targets, and are involved in a variety of physiological and pathological processes, including carcinogenesis [19], [20]. Expression profiles for miRs are tissue specific and miRs appear to be excellent biomarkers for human cancers, including breast cancer [21], [22] **.** miR profiles in cancer tissue have been associated with cancer prognosis and with tumor characteristics in several cancers, including breast cancer [23]–[25]. Several miRs have also been shown to be involved in breast cancer metastasis [26], [27]; however no study to date has investigated their role in predicting breast cancer LR. In this study, we performed miR profiling and assessed the predictive potential of miR candidate markers in the tumor tissue of breast cancer patients with and without LR.

## Results

### Characteristics of Study Population

The characteristics of the study subjects are presented in Table 1. There were no significant differences between cases with and without LR in demographic and clinical factors, such as age at diagnosis, race, histological type, tumor size, type of surgery, year of surgery and use of systemic therapy. Cases with LR had higher percentage of advanced stage (III – IV) disease (18.2% vs.6.6%) and fewer ER positive tumors (56.5% vs. 80.0%, p = 0.05) compared with those with no recurrence (Table 1).

**Table 1:** Distribution of sample characteristics by patient LR status.

Variables	Recurrent	Non-recurrent	*P* value∗
	N = 23	N = 45	
**Age at diagnosis, mean (SD)**	51.1 (13.9)	53.4 (11.4)	0.31
**Tumor size in cm, mean (SD)**	2.19 (1.17)	1.91 (1.49)	0.14
**Months of follow-up,** **mean (SD)**	83.0 (59.1)	74.7 (53.0)	0.58
**Race, N (%)**			0.41
White	12 (52.2)	29 (64.5)	
Black	6 (26.1)	6 (13.3)	
Others	5 (21.7)	10 (22.2)	
**Histological type**			0.65
Ductal carcinoma	20 (87.0)	36 (80.0)	
Duct &/or lobular carcinoma	1 (4.3)	6 (13.3)	
Others	2 (8.7)	3 (6.7)	
**Stage, N (%)**			0.26
0 - I	5 (23.8)	16 (35.5)	
II	12 (57.1)	26 (57.8)	
III - IV	4 (19.1)	3 (6.7)	
**Type of surgery, N (%)**			0.35
Lumpectomy	10 (43.5)	17 (37.8)	
Partial Mastectomy	6 (30.1)	7 (15.5)	
Total mastectomy	7 (30.4)	17 (37.8)	
Others	0	4 (8.9)	
**Year of Surgery, N (%)**			0.74
Before 1995	2 (10.0)	5 (13.9)	
1995–1999	11 (55.0)	16 (44.4)	
After 1999	7 (35.0)	15 (41.7)	
**Systemic therapy, N (%)**			
None	4 (17.4)	16 (35.5)	0.34
Radiation therapy only	3 (13.0)	8 (17.8)	
Chemotherapy only	6 (26.1)	8 (17.8)	
Both	10 (43.5)	13 (28.9)	
**E 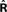 status, N (%)**			**0.05**
Positive	13 (56.5)	36 (80.0)	
Negative	10 (43.5)	9 (20.0)	

∗p-values were based on Wilcoxon rank sum test (continuous variables), chi-square or Fisher’s exact test (categorical variables) ∧estrogen receptor.

### Identification of miRs Candidates for Predicting Breast Cancer LR

Eleven out of 754 miRs showed significant differences in expression between tumors with LR and those without LR at p<0.05 level. 3 miRs were excluded due to low real-time PCR success rates (<50%) and 8 miRs were selected from these as candidates for further validation in expression levels (Table 2). None of 8 miR candidates showed high correlation (r >0.8) with any other miR candidates (Table S1).

**Table 2:** miR candidate selection and validation.

miR	Screening (N = 16)			Validation (N = 52)		
	PCR SuccessRate	LR/non-LR FoldChange	*P* value*	PCR SuccessRate	LR/non-LRFold Change	*P* value*
miR-643	100%	0.18	0.0008	75%	1.06	0.52
miR-375	97%	0.08	0.0012	100%	0.61	0.29
miR-758	89%	7.40	0.0012	85%	2.91	0.81
miR-573	100%	0.09	0.0028	56%	2.08	0.13
miR-135 b*	61%	29.9	0.0056	85%	0.89	0.59
miR-9	94%	10.6	0.019	100%	1.26	**0.0495**
miR-190 b	100%	0.11	0.019	98%	1.38	0.80
miR-328	100%	0.11	0.031	96%	0.59	0.35

* p-values were based on Wilcoxon rank sum test.

### Association of miR-9 with Breast Cancer LR

The 8 miR candidates selected were analyzed individually using the entire sample set (23 LR cases and 45 without recurrence); however, the samples used in the screening were excluded from statistical analysis in the validation step. The measurements of miRs in the screening sample set were consistent between the screening and validation studies, with significant correlation (r ranged from 0.58 to 0.89, p<0.05) between screening and validation delta Cts of all miR candidates except miR-758 (r = 0.30, p = 0.25, data not shown).

**Figure 1:**
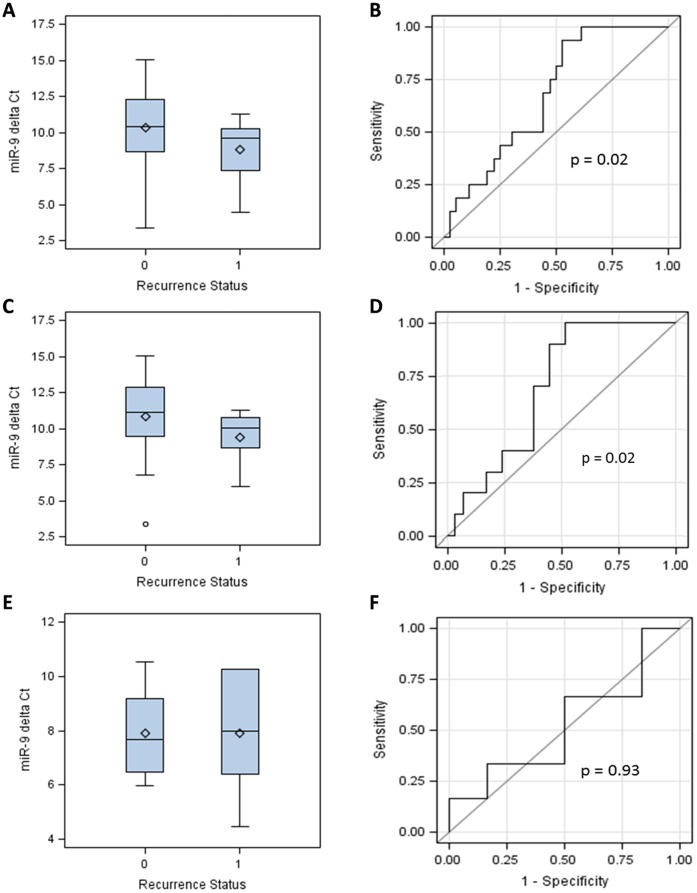
Association of miR-9 expression levels with breast cancer LR. Delta Ct values of miR-9 are compared between breast cancer patients with (recurrence status  = 1) and without (recurrence status  = 0) breast cancer LR in all tumors (panel A), and in estrogen receptor positive (panel C) and negative (panel E) tumors. A high delta Ct value indicates a low expression level. ROC curves are drawn to show the capability of miR-9 to discriminate LR in all tumors (panel B), ER positive tumors (panel D) and ER negative tumors (panel F).

miR-9 was the only miR candidate that showed significantly different expression levels between cases with and without LR (Table 2). The expression of miR-9 was significantly higher in tumors from patients with LR compared to tumors from patients without LR (average fold change  = 1.26, p = 0.0495, table 2). Distributions of delta Ct values of miR-9 were compared between cases with and without LR. The median delta Ct values of miR-9 were 10.24 (range: 3.38 to 15.02) in cases without LR and 9.60 (range: 4.45 to 11.29, p = 0.02) in cases with LR (Figure 1A). Regarding the potential of miR-9 to discriminate cases with and without LR, the AUC of ROC curve of miR-9 was computed to be 0.68 (p = 0.02), validating its value in predicting LR (Figure 1B).

As ER status is strongly associated with miR-9, the association between miR-9 and LR was further investigated in ER positive and ER negative cases separately. In ER positive cases, the median delta Ct values of miR-9 were 11.11 (range: 3.38 to 15.02) in cases without LR, and 10.04 (range: 5.96 to 11.29) in cases with LR (p = 0.02, Figure 1C). In ER negative cases, the mean delta Ct values of miR-9 were 7.67 (range: 5.98 to 10.52) in cases without LR, and 8.00 (range: 4.45 to 10.27) in cases with LR (p = 0.93, Figure 1E). Significant association with LR was observed in ER positive samples with AUC of ROC curve of 0.69 (p = 0.02, Figure 1D), but not in in ER negative cases (AUC  = 0.53, p = 0.93, Figure 1F).

**Figure 2:**
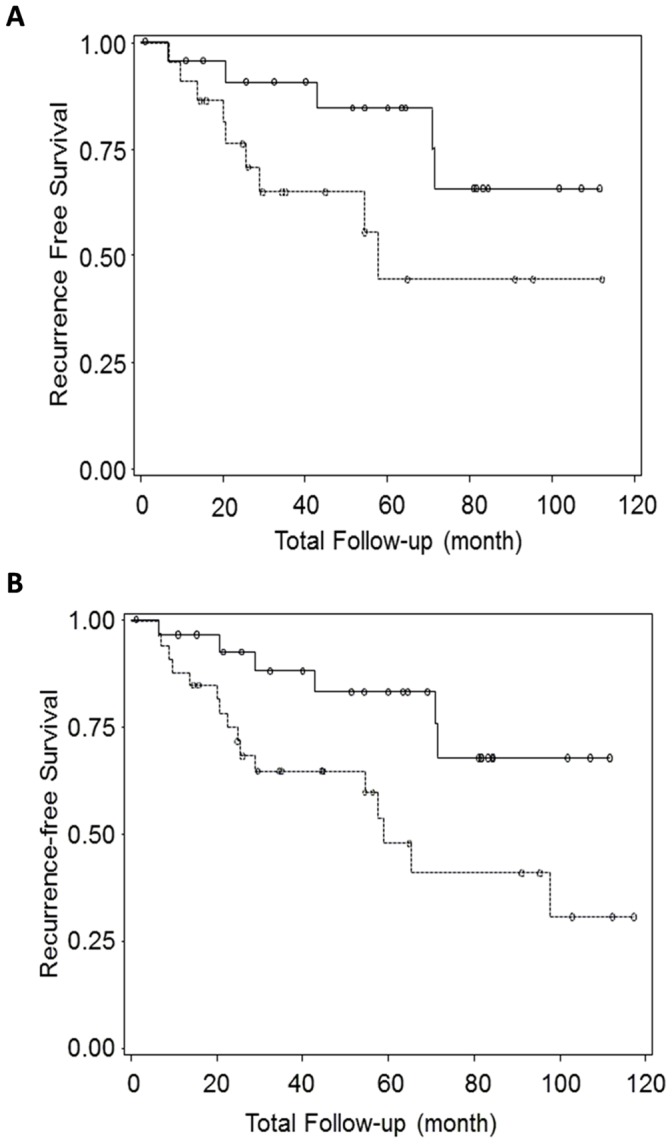
Kaplan-Meier survival curves for miR-9. Solid lines represent LR-free survival curves of breast cancer patients who had miR-9 low expression tumors in validation sample set, all cases (panel A) and ER positive cases (panel B). Dotted lines represent the patients who hadmiR-9 high expression tumors. P values are 0.08 and 0.02 for all cases (panel A) and ER positive cases (panel B), respectively.

When dichotomized using the median expression level in cases without recurrence, Kaplan-Meier analysis revealed that patients with low expression levels of miR-9 had better 10-year LR-free survival than those with high levels. 65.7% of patient with low miR-9 level did not have LR at 10 years, compared to 44.5% of patients with high miR-9 level (p = 0.08, Figure 2A). When only ER positive cases were analyzed, the 10-year LR-free survival between patients with low and high miR-9 levels showed substantial and statistically significant differences, with 67.9% 10-year LR-free survival rate in patients with low miR-9 expression compared to 30.8% 10-year LR-free survival rate in patients with high miR-9 expression (p = 0.02, Figure 2B).

### Association of miR-9 and miR-375 with Tumor ER Status

**Figure 3:**
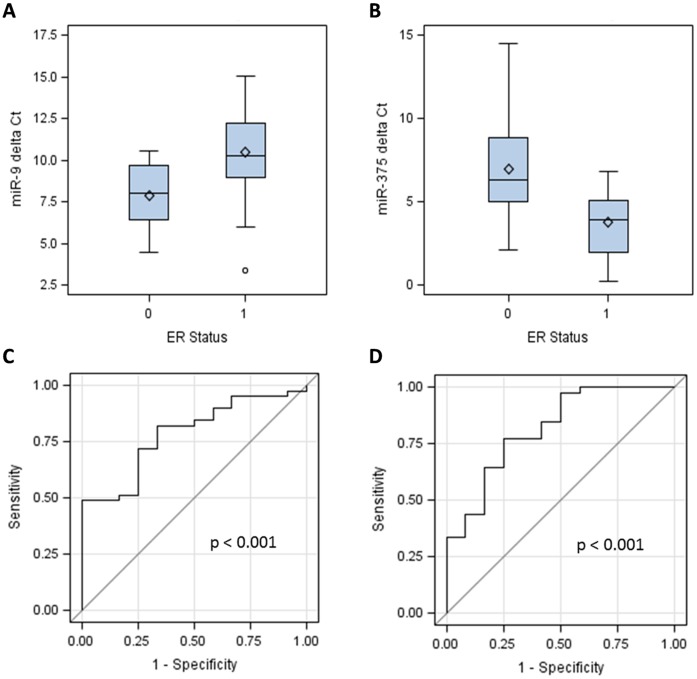
Association of miR-9 and miR-375 expression levels with tumor estrogen receptor (ER) status. Delta Ct values of miR-9 (panel A) and miR-375 (panel B) are compared between patients who had ER negative (ER status  = 0) and ER positive (ER status  = 1) tumors. A high delta Ct value indicates a low expression level. The capabilities of miR-9 and miR-375 to discriminate ER status are shown in ROC curves (panel C and D, respectively).

We examined associations between expression levels of miR candidates and other tumor, clinical, and demographic factors that were known to be associated with breast cancer LR. miR-9 expression level was significantly associated with ER status (p<0.001) and clinical stage (p = 0.03, Table 3). The mean delta Ct values of miR-9 were 7.83 (range: 4.45 to 10.52) in ER negative samples and 10.44 (range: 3.38 to 15.02) in ER positive samples (Figure 3A). No statistically significant associations between miR-9 and age at diagnosis, tumor size and histological type, year and type of surgery, and systemic therapy were found (Table 3).

**Table 3:** Association of miR-9 and miR-375 with selected patient characteristics.

	miR-9 (2^−Δct^)		miR-375 (2^−Δct^)	
Variables	median (IR)	*P* value*	median (IR)	*P* value*
**Age at diagnosis (years)**		0.26		0.69
≤50	0.13 (0.05–0.78)		3.54 (1.11–22.86)	
>50	0.08 (0.02–0.17)		5.27 (2.07–22.74)	
**Tumor size (cm)**		0.09		0.63
<1.5	0.06 (0.02–0.13)		4.81 (1.89–19.29)	
≥1.5	0.12 (0.03–0.92)		6.63 (2.07–25.15)	
**Race**		0.07		0.61
White	0.09 (0.02–0.36)		4.88 (1.71–24.04)	
Black	0.80 (0.05–1.18)		3.49 (0.18–28.95)	
Other	0.08 (0.01–0.18)		8.25 (3.10–24.04)	
**Histological type**		0.41		**0.03**
Duct carcinoma	0.10 (0.03–0.43)		3.49 (1.42–16.35)	
Duct &/or lobular carcinoma	0.05 (0.01–0.17)		6.33 (5.27–8.39)	
Others	0.09 (0.06–9.63)		29.44 (28.95–83.15)	
**Stage, N (%)**		**0.03**		0.62
0 – I	0.10 (0.04–0.44)		7.71 (2.30–23.45)	
II	0.08 (0.02–0.20)		4.88 (1.53–9.84)	
III – IV	0.82 (0.80–1.17)		3.54 (2.01–26.22)	
**Type of surgery**		0.96		**<0.01**
Lumpectomy	0.11 (0.01–1.59)		22.80 (14.53–30.89)	
Partial Mastectomy	0.08 (0.06–0.35)		2.07 (0.49–2.96)	
Total mastectomy	0.13 (0.05–0.49)		13.09(8.24–22.65)	
Others	0.09 (0.02–0.35)		3.59 (1.42–24.04)	
**Year of Surgery**		0.69		0.11
Before 1995	0.10 (0.02–0.17)		2.33 (1.10–2.96)	
1995–1999	0.11 (0.06–0.35)		7.53 (2.07–27.56)	
After 1999	0.07 (0.04–0.33)		6.63 (3.32–19.54)	
**Systemic therapy**		0.35		**0.02**
None	0.07 (0.03–0.16)		23.45 (4.76–26.83)	
Radiation therapy only	0.17 (0.08–0.78)		6.66 (2.65–14.53)	
Chemotherapy only	0.03 (0.01–0.35)		2.58 (1.42–3.59)	
Both	0.10 (0.05–0.43)		3.07 (1.03–6.63)	
**ER∧ status**		**<0.01**		**<0.01**
Positive	0.08 (0.02–0.20)		6.63(2.96–25.15)	
Negative	0.39 (0.13–1.15)		1.34 (0.22–3.09)	

* *P* values were based on Wilcoxon rank sum test (continuous variables) or Fisher’s exact test (categorical variables) ∧estrogen receptor.

Expression level of another miR candidate, miR-375, was found to be significantly associated with ER status, histological type, type of surgery and systemic therapy (Table 3). The mean delta Ct values of miR-375 were 6.79 (range: 2.14 to 14.50) in ER negative samples and 3.80 (range: 0.27 to 6.83) in ER positive samples (p<0.001, Figure 3B). Consistent with these results, miR-9 and −375 showed significant capability of predicting ER status of patients, with AUCs of 0.78 and 0.81, respectively (p<0.001, Figure 3C, D).

## Discussion

Breast cancer patients undergoing BCS are subjected to the risk of recurrence of breast cancer as well as the adverse effects caused by the standard systemic treatment after surgery. Therefore, it is highly desirable to identify biomarkers that can distinguish patients with high and low risks of recurrence. We report herein the discovery of two micro RNAs in breast tumor tissue, miR-9 and miR-375, which were associated with estrogen receptor status, one of them (miR-9) was significantly associated with local recurrence in ER positive tumors. The two miR markers were identified by a real-time PCR screening of 754 miR expression profiles followed by validation of selected miRs in 52 breast tumors, among those 16 presented LR and 36 did not.

Although this is the first report associating these miRs with estrogen receptor and LR in breast cancer, miR-9 and miR-375 have been shown to play important roles in many biological processes including carcinogenesis at different biological sites. Under physiologic conditions, miR-9 has been described as having a role in the development of the nervous system [28] and hepatocytes [29], and in the negative regulation of the acute responses of innate immunity [30]. In cell line studies, miR-9 has been observed to target junction protein E-cadherin, facilitating metastases and stimulating angiogenesis in breast cancer and HCC cells [31], [32]. Methylation and down-regulation of miR-9 was frequently observed in colorectal cancer cell lines and primary CRC tumors and associated with lymph node metastasis [33]. In addition, miR-9 is involved in the carcinogenesis of biliary tract carcinoma [34], glioblastoma [35], colorectal cancer [36], Burkitt lymphoma [37], clear cell renal cell carcinoma [38] and gastric cancers [39].

miR-375 is generally considered to be a tumor suppressor and thus is down-regulated in many types of cancers, including esophageal squamous cell carcinoma [40], head and neck squamous cell carcinoma [41], Pancreatic cancer [42], melanoma [43], and Esophageal Cancer [44]. Ectopic expression of miR-375 inhibited melanoma cell proliferation, invasion, and cell motility [43]. Conversely, ectopic expression of miR-375 is shown to repress cancer progression in pancreatic cancer [42], melanoma [43], gastric cancer [45], and liver cancer [46]. However, there are conflicting reports regarding the association between miR-375 level and cancer prognosis, suggesting a more complex and possibly cancer specific relationship. It was reported to be significantly lower in the serum of esophageal squamous cell carcinoma [47], and a 20-fold decrease of miR-375 was observed in oral and pharyngeal squamous cell carcinoma samples compared with normal control tissues [48]. In contrast, higher expression of miR-375 has been identified in tumors of prostate cancer [49], in the sputum of lung adenocarcinoma patients [50], in the serum of HBV and HBV positive HCC patients [51], and gastric cancer patients with high risk of recurrence following surgical resection [52].

Thus, in general miR-9 is associated with cancer progression while miR-375 is thought to be a cancer suppressor. The previous findings are consistent with our observations that miR-9 expression is higher in breast cancer patients with LR. The higher expression of miR-9 in cancer cells may indicate a more aggressive tumor, also suggested by the association with higher stage in our study.

In our study, both miRs were found to be significantly associated with ER status in breast cancer. Epigenetically deregulated in breast cancer, miR-375 was previously shown to form a positive feedback loop with estrogen receptor alpha in MCF-7 cells, with high expression of miR-375 in ERα-positive breast cell lines being a key driver of their proliferation [53]. This is consistent with our observation that miR-375 expression is higher in ER positive tumors. There has been no report on any possible link between miR-9 and ER status.

To our knowledge, this is the first study to report on breast tissue miRs as biomarkers of LR in breast cancer. Several previous studies have reported the discovery of miRs involved in breast cancer distant metastasis or being associated with clinico-pathological characteristics indicative of prognosis [23], [26], [27], [54]. In general, these studies identified prognostic associated miRs that were not previously found to be involved in breast cancer, possibly due to differences in experimental procedures or cell specific miRs that are usually not detected when analyzing whole tumor samples. In our study, we carefully dissected only tumor cells to be analyzed, limiting the contamination with stromal and normal adjacent cells.

In terms of experimental procedure, we used real-time PCR based methods for both screening and validation. However, discrepancies in miR measurements between these two steps could exist because a multiplex mix of primers was used in the screening while individual primer pairs were used in the validation. The use of a mixture of primers could induce potential bias due to competition for template. To monitor the effect of this factor, we repeated quantification of the 16 screening samples together with the validation samples using individual real-time PCR assay. Our results indicated that the delta Ct values of the 16 screening samples in multiplex and single real-time PCR reactions showed reasonable correlation. At the same time, the fold changes of miR candidates between LR cases and non-LR cases in the entire validation sample set were generally much smaller than those in the screening set (Table 2). This observation suggests the differences between the screening and validation results are largely due to the variation between the screening and validation sample sets rather than the differences between the assay methods used for screening and validation. The small sample size of our study could be the major contributing factor of this variation.

In summary, this study revealed that high expression of miR-9 was significantly associated with an increased risk of breast cancer LR in patients who were diagnosed with ER positive cancer. We also found that miR-9 and miR-375 were strongly associated with ER status of breast tumors. These promising data warrant further investigation to verify if the expression level of miR-9 in breast cancer cells can be a useful biomarker, in combination with other known risk factors, to identify patients at high risk of breast cancer local recurrence.

## Materials and Methods

### Ethics Statement

The study was approved by the MedStar Research Institute-Georgetown University Oncology Institutional Review Board. The requirements for informed consent from participants were waived by the Institutional Review Board because all the data and pathological specimens were previously collected and analyzed anonymously.

### Patient Population

Between 1990 and 2006, a total of 2,025 breast cancer patients who had informative follow-up data were recorded in the cancer registry database of the Lombardi Comprehensive Cancer Center (LCCC). Of these, 1,654 (81.7%) had no disease recurrence at the last contact (December, 2009), 74 (3.7%) had LR, 200 (9.9%) had distant recurrence, 45 (2.2%) were never disease free, and 50 (2.5%) had unknown type of recurrence. We included in this study the 74 patients with LR and randomly selected 148 patients who had no disease recurrence, matched on year of diagnosis (5-year interval), type of surgery (total mastectomy, partial mastectomy/segmental mastectomy, lumpectomy), and age at diagnosis (5-year interval).

The Clinical Molecular Epidemiology Shared Resources (CMESR) of the LCCC provided de-identified clinical and treatment data, including age at diagnosis, date of birth, date of diagnosis, race, type of surgery, date of surgery, disease stage, tumor size, radiotherapy and type, radiotherapy date, chemotherapy and type, chemotherapy date, tumor ER/PR status, recurrence type, recurrence date, date of first contact, date of last contact, vital status, date of death. The data were downloaded from the Cancer Registry of the LCCC and a unique study ID was assigned to each patient. All patient identifiable information was removed before the data were sent out to the study team for data analysis.

### Tumor Tissue Retrieval

Tumor blocks were available and retrieved by the Histopathology and Tissue Shared Resources of Lombardi Comprehensive Cancer Center for 112 (50.5%) of the 222 patients selected for the study. Eight serial 5-micron and four 20-micron sections were cut from each block and the first section was H&E stained. One 20-micron section was used for total RNA isolation. The study pathologist (BK) examined and marked tumor areas on all H&E slides. Only 68 tumors containing more than 70% tumor cells were used for this study. All the tissue sections were labeled with a unique study ID number.

### RNA Isolation and Reverse Transcription

Total RNA was extracted from dissected FFPE breast tissue samples using the RecoverAll Total Nucleic Acid Isolation Kit (Ambion, Grand Island, NY) according to the manufacturer’s instructions. RNA quantity was assessed with the NanoDrop1000 (Thermo Fisher Scientific, Inc., Waltham, MA). The total RNA yield ranged from 0.12 to 17.27 µg with an average of 2.45 µg. For each sample, 1 µl total RNA was used for reverse transcription to create cDNA template for Real-Time PCR using Taqman Small RNA Assays kit (Applied Biosystems, Carlsbad, CA).

### miR Screening and Validation

miR expression profiling was performed using RNA samples isolated from 16 tumors (8 with LR and 8 with no LR) utilizing the TaqMan Array Human MicroRNA Set v3.0 (Applied Biosystems) as described by the manufacturer. For validation, total RNA samples from tumor tissues of 16 cases with LR and 36 without LR were isolated and the expression level of individual miR was analyzed using Taqman Small RNA Assays (Applied Biosystems) according to manufacturer’s instructions. The mean delta Cts of triplicate real-time PCR amplifications were used for statistical analysis. The comparative delta Ct values were used as the relative quantification of miRs, using the U6 small RNA for normalization (delta Ct  =  Ct^miRs^ – Ct^U6^). Ct values of 9 out of the total 544 real-time PCR reactions (1.7%) were rejected due to a coefficient of variance larger than 5% among triplicates. Technical staffs that performed the miR assays were blinded to the recurrence status of the samples.

### Statistical Analysis

Wilcoxon rank-sum tests, chi-square, or Fisher’s exact tests were used to examine the differences between LR and non-LR cases in continuous and categorical variables, respectively. ROC curves were generated by plotting sensitivity vs. (1-specificity), and AUC was calculated and used to assess the possible predictive value of each miR in classifying patients into recurrent or non-recurrent status. Kaplan-Meier analysis for recurrence-free survival was estimated for patients according to their relative amount of individual miRs in the tumor tissue and compared using Log-rank test. Delta Ct values was dichotomized as low/high using the median value in non-recurrent cases as the cutoff point. P-values were two-sided and considered significant if p<0.05. All analyses were performed using SAS software, version 9.3 (SAS Institute, Inc., Cary, NC).

## Supporting Information

Table S1 **Correlations among miR candidates.**
(DOCX)
