# Supplementary material for: MicroRNA-9 as Potential Biomarker for Breast Cancer Local Recurrence and Tumor Estrogen Receptor Status
Source: PLoS One. 2012 Jun 18;7(6):e39011. doi: 10.1371/journal.pone.0039011 (PMC3377597; doi:10.1371/journal.pone.0039011)
Supplement: Table S1 — Correlations among miR candidates. (DOCX) [file pone.0039011.s001.docx]

**Supplemental Table S1. Correlations among miR candidates**

|  | miR-9 | miR-328 | miR-375 | miR-758 | miR-135b* | miR-643 | miR-573 | miR-190b |
| --- | --- | --- | --- | --- | --- | --- | --- | --- |
| miR-9 | 1.00 | -0.34 | -0.06 | 0.62 | 0.33 | 0.05 | -0.54 | 0.28 |
| miR-328 | -0.34 | 1.00 | 0.44 | -0.20 | -0.52 | 0.01 | 0.31 | 0.34 |
| miR-375 | -0.06 | 0.44 | 1.00 | -0.36 | -0.35 | 0.02 | -0.14 | 0.45 |
| miR-758 | 0.62 | -0.20 | -0.36 | 1.00 | 0.33 | 0.03 | -0.46 | 0.07 |
| miR-135b* | 0.33 | -0.52 | -0.35 | 0.33 | 1.00 | 0.09 | -0.43 | -0.18 |
| miR-643 | 0.05 | 0.01 | 0.02 | 0.03 | 0.09 | 1.00 | 0.00 | 0.51 |
| miR-573 | -0.54 | 0.31 | -0.14 | -0.46 | -0.43 | 0.00 | 1.00 | -0.10 |
| miR-190b | 0.28 | 0.34 | 0.45 | 0.07 | -0.18 | 0.51 | -0.10 | 1.00 |
